# Virtual Supervision in a Family Medicine Residency Program: Lessons from the Early Stages of a Pandemic!

**DOI:** 10.15694/mep.2021.000024.1

**Published:** 2021-01-28

**Authors:** MaryBeth DeRocher, Fok-Han Leung

**Affiliations:** 1 Department of Family & Community Medicine

**Keywords:** Postgraduate Medical Education, Family Medicine, Virtual Supervision

## Abstract

This article was migrated. The article was marked as recommended.

The landscape of postgraduate education in a family medicine residency changed abruptly with the onset of the pandemic in March 2020. The early weeks and months of the pandemic have highlighted some short-fallings in virtual supervision that were not anticipated based upon our previous ways of teaching. In order to support the essential components of family medicine postgraduate teaching, curricular and program structural changes are required (which will likely translate into further iterative improvements). This opinion piece highlights some early changes in our large Canadian Family Medicine Residency Program, combining our early reflections on virtual supervision, the practicalities of on-the-ground teaching, and the existing concepts from the literature supporting effective medical teaching.

## Introduction

An incredible overhaul of clinical activities occurred at the outset of the pandemic. Similarly, we pivoted hard to adapt our Family Medicine Residency Program to the new realities. As the largest clinical training site within the University of Toronto’s Family Medicine Program, our cohort includes 38 - 40 family medicine residents in the two-year program and is home to close to 80 family physicians and over 200 interprofessional team members.

In March 2020, we quickly flipped our typical resident learning experiences into “virtual” mirror images. Our residents had traditionally run their own independent clinics, seeing approximately 8 - 10 patients per half-day. During most of these clinics, family medicine preceptors simultaneously ran their own ambulatory clinics with an several 15-minute breaks inserted for supervision and review. The Pandemic-evoked version of this looked quite similar: resident schedules of virtual appointments at the same volumes, with parallel supervision. We celebrated this transition and applauded ourselves for our adaptability. While we bemoaned the newness of virtual patient care, we didn’t consider the drastic changes we needed to make in our educational approach.

Our conversion to a copy-and-paste virtual supervision approach was reflexive, in response to the rapidly changing times. However, looking to the literature, there is a paucity of data to support how best to facilitate virtual supervision in a postgraduate education program, even if we had had the time to do it. Unbeknownst to us, we were falling prey to the previously described fallacies of medical education in an increasingly virtual world ( Cameron, Ray and Sebesan, 2015). Some of the principles that traditionally lent to an effective learning experience for residents were inadvertently lost: accessibility of supervisor ( Wearne *et al.,* 2012), ability for learners to observe their preceptors participating in routine patient care ( Rietmeijer *et al*., 2018), and commitment of supervisors to their teaching role as a priority amidst patient care ( Scheepers *et al.,* 2015).

Within the first four weeks, we noted some important trends that have led to a change in our supervisory practice. Based upon our early learnings, we present our reflections on the required changes we will need to implement for successful virtual supervision.

## Early Learnings

### 1. We need to build in opportunities for debrief and review

Previous in-person supervision allowed for organic discussions about logistics and scheduling. However, off-site clinical supervision risks missed connections and assumptions about how and when to touch base. Our reflections on the first few months of virtual supervision has taught us that we need protected time to review the logistics that we previously took for granted. We needed to ensure that there was a protected time period at the beginning of the clinic to promote the discussions of how cases will be reviewed, when they will be reviewed, and when they should be reviewed. Similarly, we need to build in the tools that we were all newly using to promote teaching: links to password protected videoconferencing tools were being included as staples in the EMR schedule.

### 2. Networking platforms/technologies need to be leveraged not just for patient care but for clinical teaching

While we grappled with how to use tools like Zoom, OTN, and phone conferencing for patient care, we realized that they had a separate identity in the world of Family Medicine resident teaching. We needed to reframe these tools as options to both review cases, as well as to be involved in indirect observation of patient encounters. Perhaps most important is the acceptance of these tools as the conduit to preceptor accessibility, promoting the teacher-resident relationship (Wearne *et al.,* 2018).

For us, the early days of using these tools were clumsy. We realized that the logistics of these tools need support from those who understand them, leveraging these experts to create quick one-pager guidelines for clinical teachers who are less IT-adept. These observation tools will be especially important in the coming months as we welcome new Family Medicine residents into clinic and need to understand their level of competence with closer supervision. They will also be important tools in facilitating opportunities for residents to observe their preceptor’s approach to patient care as highlighted in the literature ( Rietmeijer *et al*., 2018).

### 3. Traditional volumes of patients for Family Medicine residents in ambulatory clinics need to be adjusted

In our program, we have traditionally upheld clinical volumes as an important facet of training in order to breed diversity, breadth, and time management. However, changes from the pandemic began to limit encounters due to our protected debrief/review and the initial fumbling with tech use. It was also realized that Family Medicine residents are using their virtual assessment skills, without the necessary in-person experience that we as preceptors are relying on from (variable) years of practice. In our opinion, Family Medicine residents need more hands-on support to enhance their learning (and ensure appropriate patient care) with virtual encounters.

Whereas residents had previously gained competence from volumes of patient exposure, we now needed to look to more intensive preceptor support to cement their learning. This takes time and results in a need for a lighter schedule. As a result, our residents are actually working off their preceptor’s schedule to work more closely with a smaller volume of patients (currently 6 patients per half day).

In-person clinical appointment volumes for residents have also be changed in response to the added time for donning and doffing of PPE and the continuous the cleaning of patient rooms, the resident schedule became much more lightly booked.

### 4. Traditional volumes of patients for Family Medicine preceptors in ambulatory clinics need to be adjusted

While we were doing remote supervision in the early days of the pandemic with our own booked clinics, we likely weren’t doing it well. The numerous reasons for this are described in above points 1 - 3. Additionally, the balancing act of fielding calls from family medicine residents between our own virtual visits resulted in an intangible level of fragmentation of clinical care and teaching. As noted above, the nature of virtual visits likely requires the more intensive support of a preceptor to be effective in both teaching and clinical care. It became apparent to us that to teach virtually (and to do it well), we needed to scale back on patient visits during supervision days, reflective of the commitment we expect for our clinical teachers ( Scheepers *et al.,* 2015). As mentioned previously, our preceptors and residents are working together off of a single patient appointment schedule and volumes have been reduced.

### 5. Assessment in a family medicine postgraduate program needs to shift in response to virtual supervision

Family Medicine Resident supervisors in our program have traditionally used written field notes to document pearls of feedback based on competencies. Internal benchmarks (for field note frequency) have been suggested, but prior to the pandemic, field notes were written for residents on the minority of clinical encounters. Now, the reduced clinical volumes demand a shift in field note provision; written feedback (via fieldnotes) should be provided after the majority of patient encounters. This will allow supervisors to fine tune the teaching which is necessary when there are lower volumes. We will also need to give more feedback in response to other facets of virtual patient care (ex: residents emailing and using secure messaging with patients).

## Conclusion

The COVID pandemic is a time of immense change, both in clinical care and medical education. We have iteratively improved our virtual care. We must now also iteratively improve our virtual teaching supervision. These five tips are initial learnings to support early findings. However, a dedicated understanding with a scholarly lens will be required to product best practices for virtual supervision in medical education.

## Take Home Messages

• Virtual supervision in postgraduate medical education cannot simply be the mirror image of traditional in-person supervision
• Virtual supervision requires protected time for debrief and review
• Networking platforms and technology need to be leveraged for clinical teaching (in addition to clinical activities for virtual care)
• Traditional volumes for both residents and teachers need to be adjusted for virtual supervision
• Assessment methods in postgraduate education need to encompass elements of virtual care and supervision
• Best Practice Guidelines on Virtual Supervision and Teaching need further development

## Notes On Contributors

**Dr. MaryBeth DeRocher** is a family physician at the St. Michael’s Hospital Department of Family & Community Medicine, University of Toronto.

**Dr. Fok-Han Leung** is a family physician at the St. Michael’s Hospital Department of Family & Community Medicine, University of Toronto.
