# Supplementary material for: Virtual Supervision in a Family Medicine Residency Program: Lessons from the Early Stages of a Pandemic!
Source: MedEdPublish (2016). 2021 Jan 28;10:24. [Version 1] doi: 10.15694/mep.2021.000024.1 (PMC10939586; doi:10.15694/mep.2021.000024.1)
Supplement: Peer review history

# Reviewer report: Reviewer response for version 1

**DOI:** 10.21956/mep.18857.r26888

**Published:** 2021-02-01

**Authors:** David Bruce

**Affiliations:** 1 None

This review has been migrated. The reviewer awarded 4 stars out of 5 In this timely and interesting paper, the authors share their experiences of the rapid changes that they have made to their family medicine residents teaching programme in response to the pandemic. These are early observations and recommendations, but are clearly presented and justified. Reading this paper confirmed to me the complexity of teaching and the importance of each of Harden’s 8 roles of a teacher. I think the authors demonstrate that simply flipping from a well-designed teaching programme to an identical virtual programme, while an expedient way to continue their teaching programme, does need review and modifications in light of their experiences. The suggested areas for review – more time for teaching, considering best use of IT platforms, less case load and tailored feedback – are all excellent steps. As the authors state more research is needed and I would be interested to read the views of their residents and teachers. This paper should be read by all involved in the design of teaching programmes and will be of interest of all teachers.

Reviewer Expertise:

NA

No decision status is available

# Reviewer report: Reviewer response for version 1

**DOI:** 10.21956/mep.18857.r26889

**Published:** 2021-01-29

**Authors:** Balakrishnan Nair

**Affiliations:** 1 Centre for Medical Professional Development and University of Newcastle

This review has been migrated. The reviewer awarded 4 stars out of 5 Thank you for asking me to review this paper. I enjoyed reading this opinion piece. All educators are struggling and improvising on the run with virtual teaching ,and will benefit from this article. The authors set up the virtual consults for the trainees and then set up parallel supervision. Their reflection on this process led to some refinement to the program. Some of these changes are the more protected time to set the goals, the teacher’s need for IT upskilling and refining the volume load. They also learned to give feedback in the new setting Teaching is all about learning; some more input and reflections from the learners would enrich this study.

Reviewer Expertise:

NA

No decision status is available
